# Supplementary material for: The RepID–CRL4 ubiquitin ligase complex regulates metaphase to anaphase transition via BUB3 degradation
Source: Nat Commun. 2020 Jan 7;11:24. doi: 10.1038/s41467-019-13808-9 (PMC6946706; doi:10.1038/s41467-019-13808-9)
Supplement: Supplementary file 1 — Supplementary Information [file 41467_2019_13808_MOESM1_ESM.pdf]

Supplementary Information

The RepID-CRL4 ubiquitin ligase complex regulates metaphase to anaphase transition via BUB3 degradation

Jang et al.

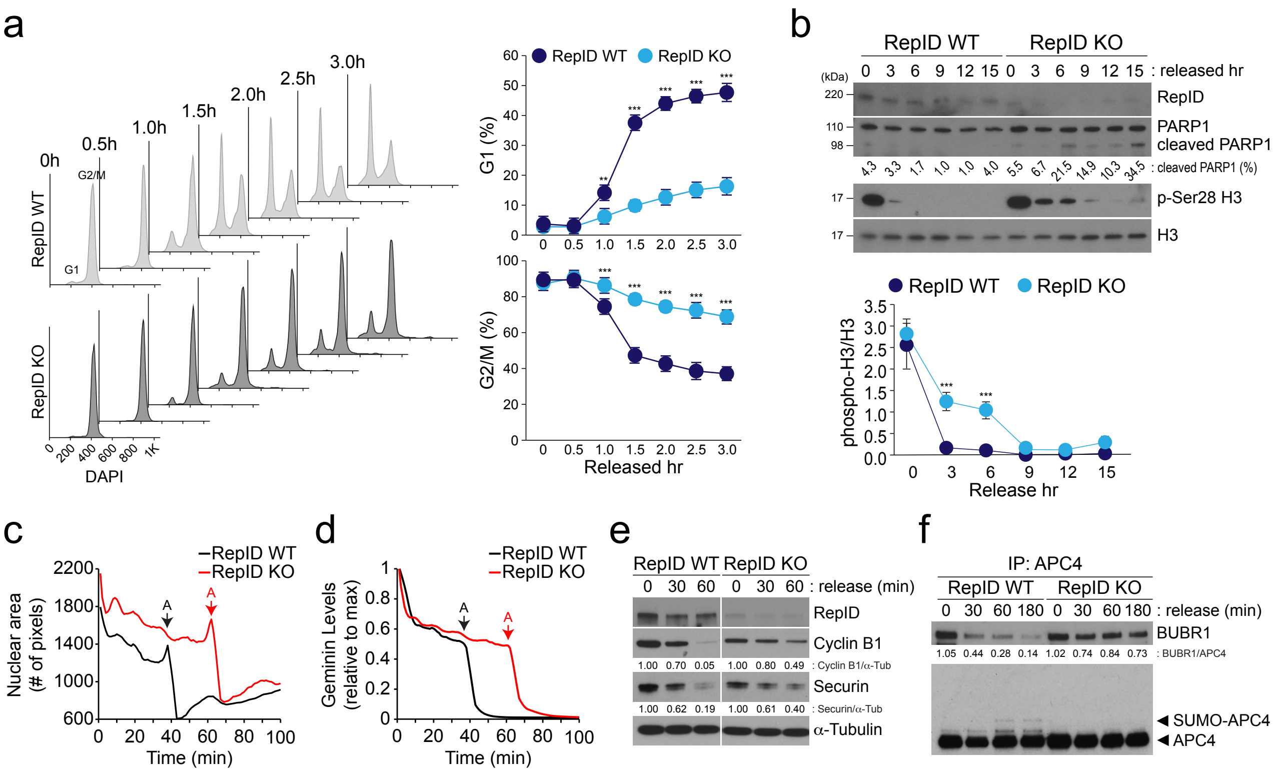

Supplementary Figure 1. RepID roles in mitotic exit. (**a**) HCT116 RepID WT and KO cells were synchronized by nocodazole for 16 hours, released in fresh media and collected every 30 minutes, followed by flow cytometry (left panel). Percentage of cells in G1 and G2/M fractions in histogram (right panel). Error bars represent standard deviations from three independent experiments (**p-value < 0.01, ***p < 0.001, Student's t-test). (**b**) HCT116 RepID WT and KO cells were synchronized by nocodazole, released in drug-free media and collected every 3 hours, followed by immunoblot analysis (upper panel). Relative intensity of phosphorylated histone H3 normalized by the intensity of the histone H3 signal (bottom panel). Error bars represent standard deviations from three independent experiments (***p-value < 0.001, Student's t-test). (**c,d**) Tracking of the nuclear area (H2B-mTurquoise)(**c**) and APC-degron (mCherry-geminin)(**d**) as in Fig. 1d. Anaphase initiation is indicated as "A". (**e**) APC/C activation is delayed in mitotic-released RepID KO cells. HCT116 RepID WT and KO cells were synchronized by nocodazole, released in fresh media and collected as indicated. Cyclin B1 and Securin protein levels were analyzed by immunoblotting. The numbers under the panels represent the intensity ratios for each protein normalized by the intensity of the signal at zero minutes after normalization by α-Tubulin signal. (**f**) APC/C is slowly dissociated from SAC in RepID KO cells during mitosis. Synchronized HCT116 RepID WT and KO cells were released in fresh media, collected at indicated times, and immunoprecipitation was performed using APC4 antibody. Co-precipitated BUBR1 was analyzed by immunoblotting and normalized to APC4. This figure is related to Fig. 1. Source data are provided as a Source Data file.

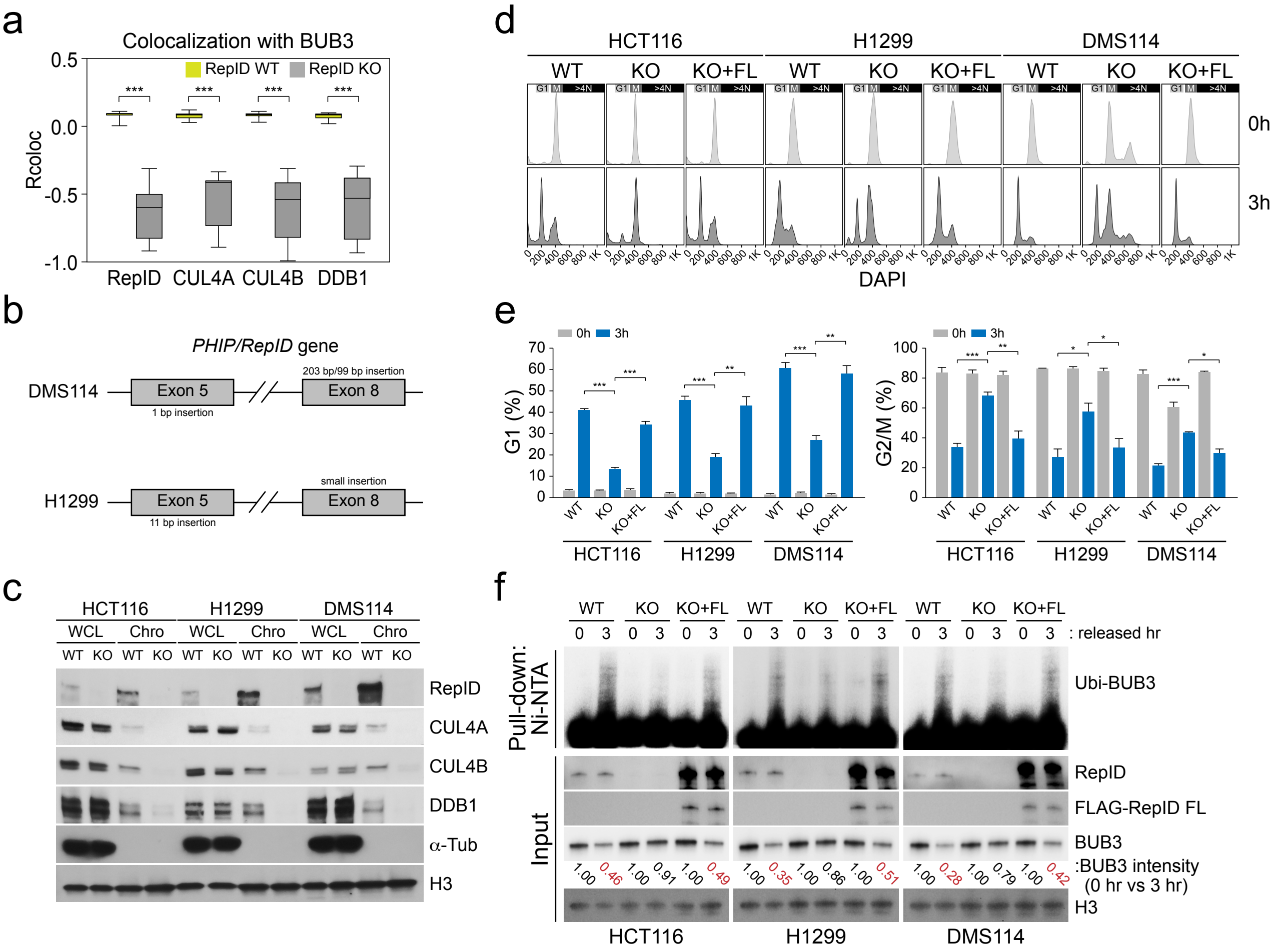

Supplementary Figure 2. RepID-CRL4 ubiquitinates BUB3 during mitosis. (**a**) Pearson's correlation coefficients (n=10) between BUB3 and RepID, CUL4A, CUL4B or DDB1 and p-values were calculated using a two-tailed t-test (***p-value<0.001). (**b**) Schematic representing RepID KO generation by CRISPR-Cas9 gene editing system in H1299 and DMS114 cells. (**c**) Chromatin-bound CRL4 components were analyzed by immunoblotting in RepID WT and KO in HCT116, H1299 and DMS114 cells. (**d**) RepID WT, KO and reconstituted RepID FL in KO background in three cell lines were synchronized by nocodazole for 16 hours, released in fresh media and collected after 3 hours, followed by flow cytometry. (**e**) Percentage of cells in G1 and G2/M fractions in (d). Error bars represent standard deviation from three independent experiments (*p-value<0.05, **p<0.01, ***p < 0.001, Student's t-test). (**f**) HIS-tagged ubiquitin plasmid was transiently transfected into the indicated cell lines. Synchronization was carried out with nocodazole. Cells were released in fresh media for 3 hours, and his-tagged proteins were isolated on Ni-NTA beads followed by immunoblot analysis. This figure is related to Fig. 3. Source data are provided as a Source Data file.

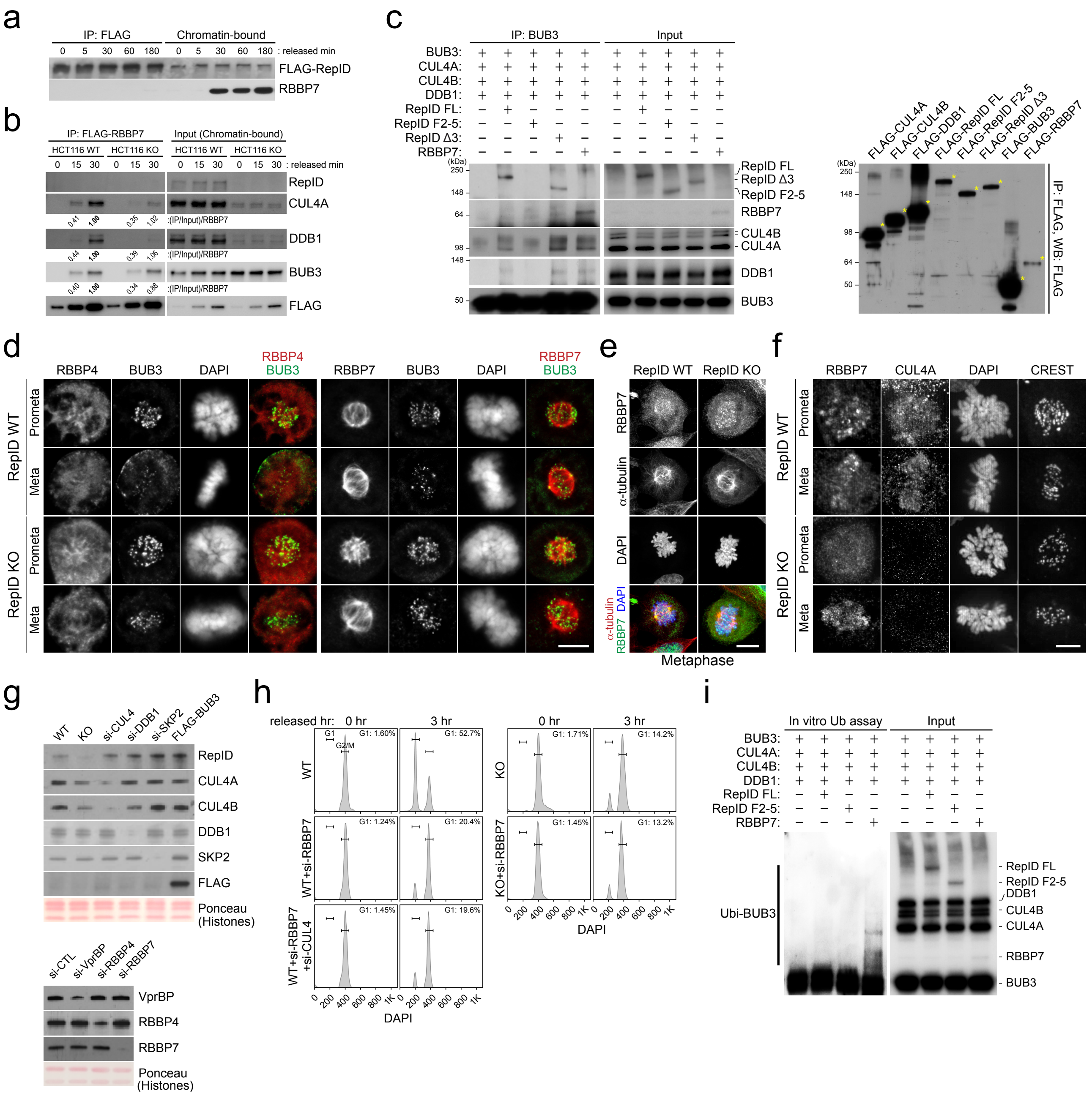

Supplementary Figure 3. RBBP7 is incorporated into CRL4 for BUB3 ubiquitination during metaphase. (**a**) RBBP7 does not bind RepID. HCT116 cells with transiently transfected FLAG-RepID FL were released from mitotic block as indicated times. Co-precipitated RBBP7 using chromatin fractions was analyzed by immunoblotting. (**b**) FLAG-tagged RBBP7 was transiently transfected in HCT116 WT and KO cells. Cells were released from mitotic block, and FLAG-RBBP7 was used for immunoprecipitation of chromatin-bound fractions followed by immunoblotting using RepID, CUL4A, DDB1 and BUB3 antibodies. Binding ratios were calculated by normalizing to the input and the precipitated RBBP7 level. (**c**) Each purified protein was mixed and immunoprecipitated with antibodies directed against BUB3, followed by immunoblotting using FLAG antibody (left panel). Confirmation of purified proteins by immunoblotting analysis (right panel). Asterisks indicate each purified protein. (**d-f**) RBBP7 localizes to both mitotic spindles and kinetochores. (**d**) Immunofluorescence analysis was performed using RBBP4 or RBBP7 with BUB3 antibodies in HCT116 RepID WT or KO cells. Scale bar: 10 μm. (**e**) HCT116 RepID WT and KO cells were used for immunofluorescence analysis using anti-RBBP7 and anti-α-tubulin antibodies. Scale bar: 10 μm. (**f**) Colocalization between RBBP7 and CUL4A increases in RepID WT cells during metaphase. Immunofluorescence analysis using RBBP7, CUL4A and CREST antibodies in HCT116 RepID WT or KO cells. Scale bar: 10 μm. (**g**) Verification of knockdown mediated by siRNA-CUL4, DDB1, SKP2, VprBP, RBBP4 or RBBP7 or overexpression of FLAG-tagged BUB3. Ponceau staining was used for loading control. (**h**) HCT116 RepID WT or KO cells were transfected with indicated siRNA, synchronized by nocodazole for 16 hours, released for 3 hours, and cell cycle analysis was carried out using flow cytometry. (**i**) In vitro ubiquitination assay was performed using purified proteins, followed by immunoblotting with BUB3 antibody. This figure is related to Fig. 4. Source data are provided as a Source Data file.

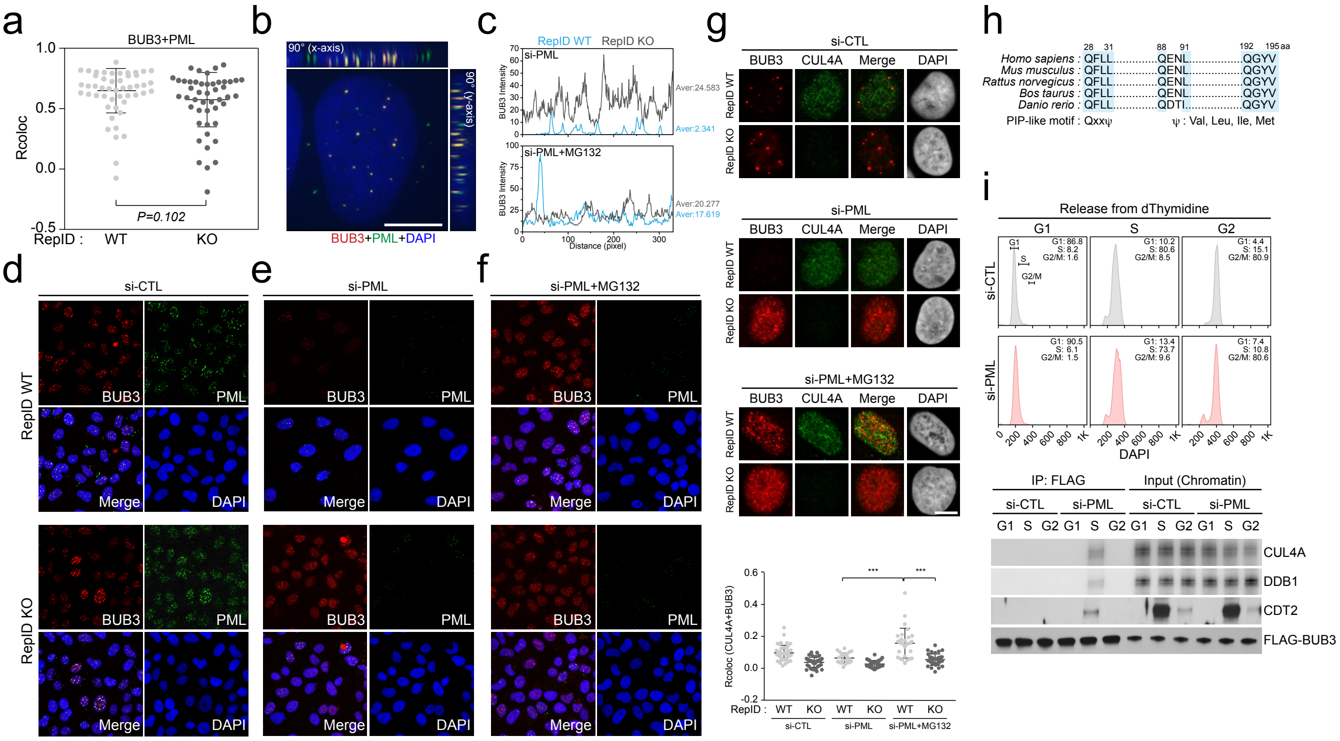

Supplementary Figure 4. PML protects BUB3 from degradation by CRL4-CDT2 during interphase. (**a**) Quantification of colocalization between BUB3 and PML in U2OS RepID WT and KO cells by Pearson's correlation coefficients (WT; n=49, KO; n=47). (**b**) Orthogonal sections from Fig. 6b. Scale bar: 10 μm. (**c**) BUB3 intensity profiles of foci as in Fig. 6d,e. (**d-f**) Different patterns and levels of endogenous BUB3 in RepID WT or KO cells with/without siRNA-PML or MG132. (**g**) BUB3 is colocalized with CUL4A in MG132-treated PML-deficient RepID WT cells. U2OS RepID WT and KO cells were transfected with control siRNA or siRNA-PML, incubated with MG132, and immunofluorescence analysis was performed using anti-BUB3 and anti-CUL4A antibodies after pre-extraction. Quantification of colocalization ratio between BUB3 and CUL4A by Pearson's correlation coefficients (***p-value < 0.001). (**h**) Highly conserved PIP-like motif in BUB3 across species. (**i**) HCT116 RepID WT cell was transfected with FLAG-tagged BUB3 plasmid together with siRNA-CTL or siRNA-PML, synchronized by double thymidine, released with fresh medium. Cell cycle for G1, S and G2 phases were verified by flow cytometry (upper panel), and chromatin fractions were used for immunoprecipitation by FLAG antibody. Co-precipitated proteins were analyzed by immunoblotting (bottom panel). This figure is related to Fig. 6. Source data are provided as a Source Data file.

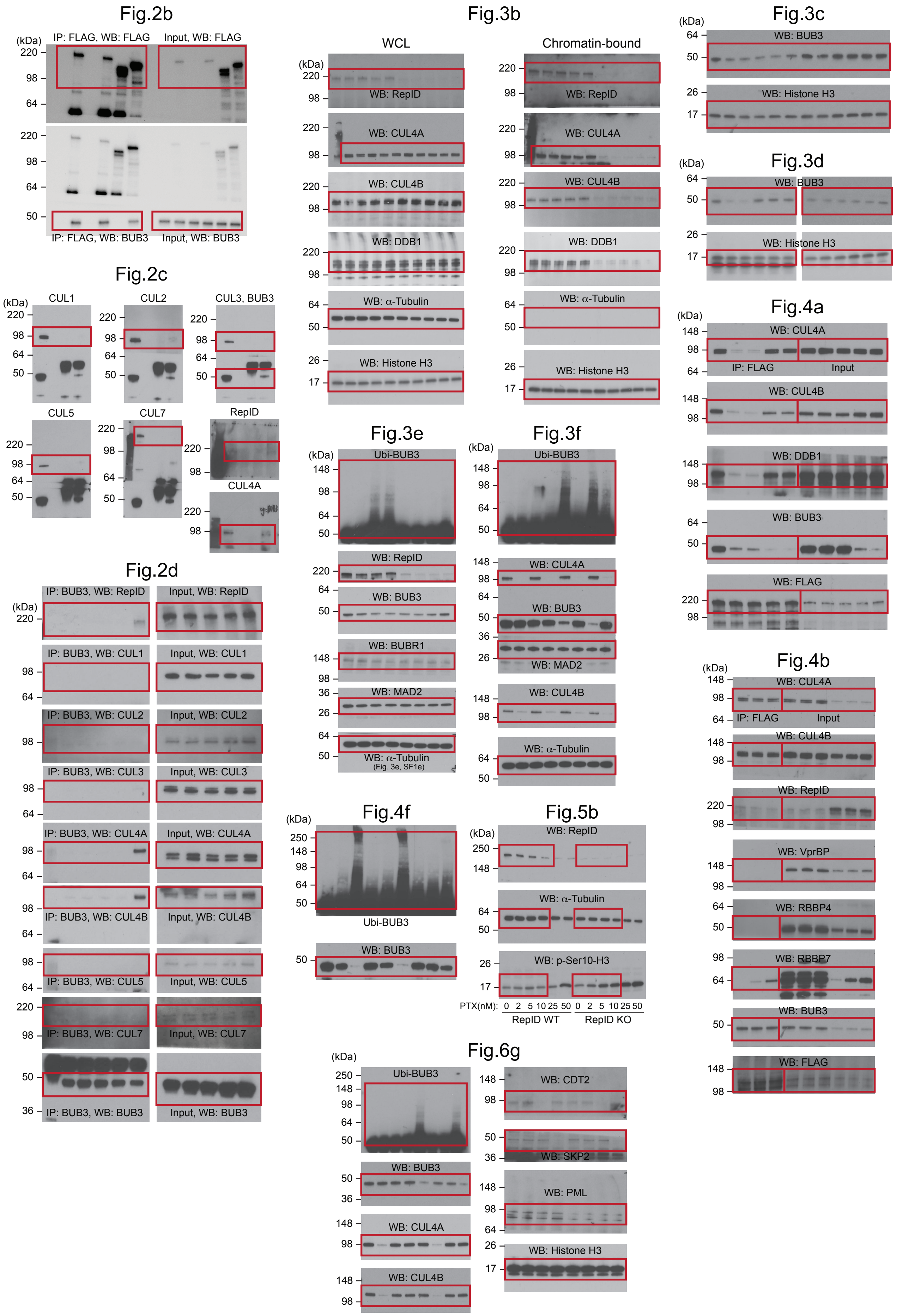

Supplementary Figure 5. Unprocessed original scan files for immunoblot analysis.

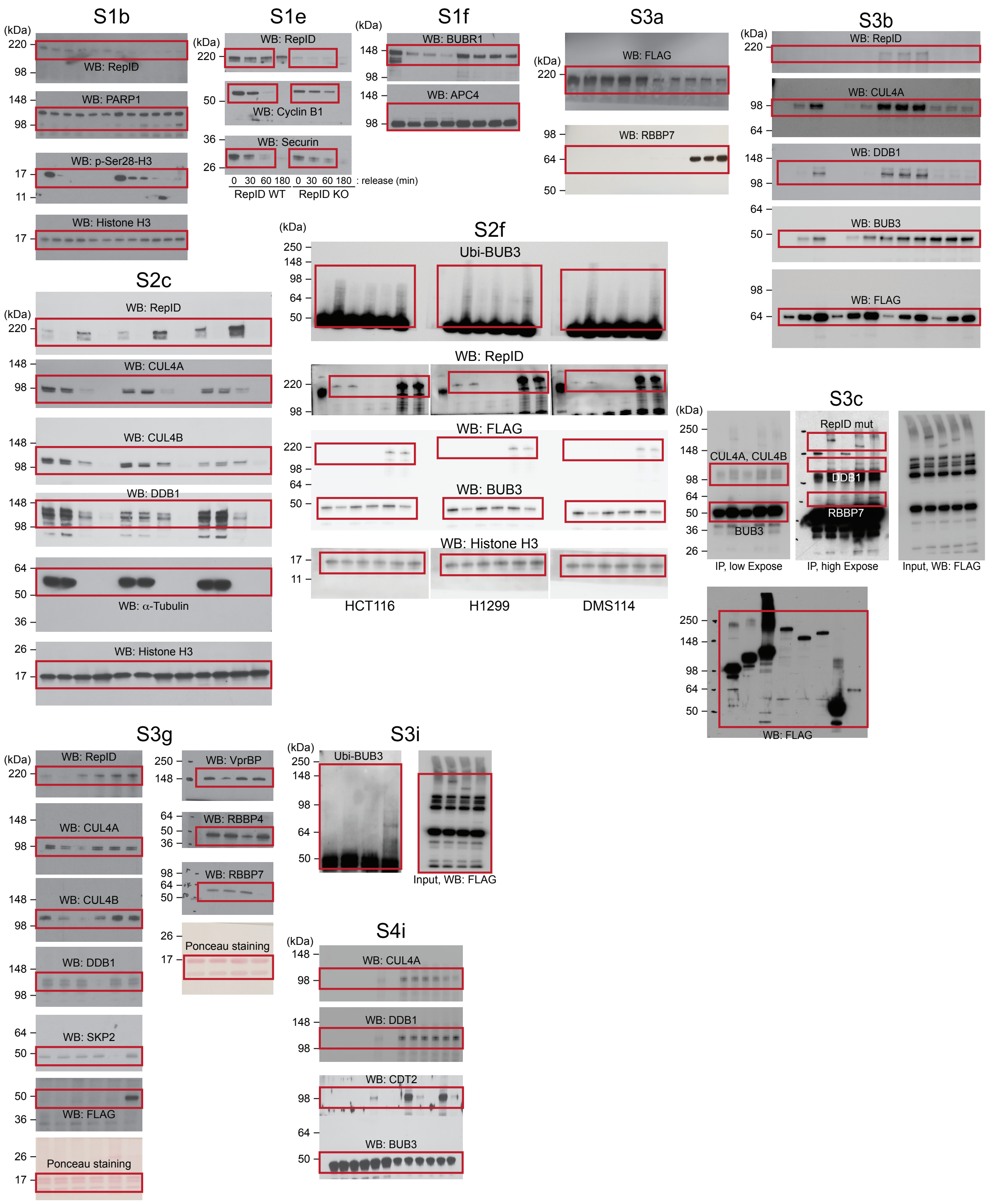

Supplementary Figure 6. Unprocessed original scan files for immunoblot analysis.
